# The Annals of Surgery

**Published:** 1890-03

**Authors:** 


### THE ANNALS OF SURGERY. Edited by L. S. Pilcher, A. M., M. D., and C. B. Keetley, F. R. C. S. J. H. Chambers & Co., 914 Locust St., St. Louis.

The February number of this excellent periodical is before us. Among its interesting contents is a case of traumatic cerebral abscess relieved by a capital operation; a hemorrhagic cyst in the brain relieved similarly. A remarkable case of cirsoid aneurism of the scalp obliterated by multiple ligatures; a rare case of dislocation of the patella beneath the inter-condyloid groove of the femur; the use of cocaine in surgery; a case of cortical epilepsy following penetrating wound of the skull, relieved by trephining, one year after injury; resection of the third branch of the trifacial nerve, and many others. It is a good journal to take with THE HOMŒOPATHIC PHYSICIAN, as it gives the surgical information that has to be excluded from our pages by reason of our devotion to the department of materia medica.

